# ADULT EDUCATION AND TRAINING IN CANADA: OPPORTUNITIES, FUNDING, AND GENDER GAPS

**DOI:** 10.1093/geroni/igz038.012

**Published:** 2019-11-08

**Authors:** A Katherine Harrington, Phyllis Cummins

**Affiliations:** Scripps Gerontology Center, Miami University, Oxford, Ohio, United States

## Abstract

Labor force participation rates for middle-aged and older Canadians have increased substantially over the past two decades, with increases for women outpacing men. Given the importance of adult education and training (AET) to stay competitive in later career, we used a mixed methods approach to examine gender differences. Our analysis of the 2012 Program for the International Assessment of Adult Competencies (PIAAC) data indicated that, for ages 55-65, rates of AET participation are similar for both men and women. However, women are less likely than men to have AET funded by their employers. Findings suggest that women are more likely to need alternate funding sources for AET, such as other organizations or through self-funding. In addition, our review of literature, policy-related documents, and key informant interviews identified possible changes in policies and practices for the promotion of AET for middle-aged and older Canadians.

